# Inactivation of *Anopheles gambiae* Glutathione Transferase *ε*2 by Epiphyllocoumarin

**DOI:** 10.1155/2016/2516092

**Published:** 2016-01-26

**Authors:** Patience Marimo, Rose Hayeshi, Stanley Mukanganyama

**Affiliations:** ^1^Department of Biochemistry, University of Zimbabwe, Mt. Pleasant, Harare, Zimbabwe; ^2^Preclinical Drug Development Platform, North West University, Potchefstroom Campus, Private Bag X6001, Potchefstroom 2520, South Africa

## Abstract

Glutathione transferases (GSTs) are part of a major family of detoxifying enzymes that can catalyze the reductive dehydrochlorination of dichlorodiphenyltrichloroethane (DDT). The delta and epsilon classes of insect GSTs have been implicated in conferring resistance to this insecticide. In this study, the inactivation of* Anopheles gambiae* GST*ε*2 by epiphyllocoumarin (Tral 1) was investigated. Recombinant AgGST*ε*2 was expressed in* Escherichia coli* cells containing a pET3a-AGST*ε*2 plasmid and purified by affinity chromatography. Tral 1 was shown to inactivate GST*ε*2 both in a time-dependent manner and in a concentration-dependent manner. The half-life of GST*ε*2 in the presence of 25 *μ*M ethacrynic acid (ETA) was 22 minutes and with Tral 1 was 30 minutes, indicating that Tral 1 was not as efficient as ETA as an inactivator. The inactivation parameters *k*_inact_ and *K*_I_ were found to be 0.020 ± 0.001 min^−1^ and 7.5 ± 2.1 *μ*M, respectively, after 90 minutes of incubation. Inactivation of GST*ε*2 by Tral 1 implies that Tral 1 covalently binds to this enzyme* in vitro* and would be expected to exhibit time-dependent effects on the enzyme* in vivo*. Tral 1, therefore, would produce irreversible effects when used together with dichlorodiphenyltrichloroethane (DDT) in malaria control programmes where resistance is mediated by GSTs.

## 1. Introduction

Glutathione transferases (GSTs; EC 2.5.1.18) belong to a large group of detoxification enzymes. These enzymes catalyze the conjugation of reduced glutathione (GSH; *γ*-L-glutamyl-L-cysteinylglycine) to xenobiotic compounds such as drugs, herbicides, and insecticides [1]. Since their discovery in 1961 [2], the glutathione transferases have come to be recognized as one of the most important families of enzymes to carry out detoxification of xenobiotics and endogenous electrophilic compounds. Most organisms have GSTs belonging to multiple classes, suggesting differing catalytic activities to accommodate the wide range of substrate specificities [3]. To date, little is known about the endogenous substrates of mosquito GSTs. Studies on mosquito GSTs have focused on their role in insecticide metabolism. Elevated levels of GST activity have been associated with resistance to all the major classes of insecticides [4]. GSTs exist in insects, mammals, and plants. Most of them are cytosolic, although microsomal or mitochondrial forms also exist. GSTs are dimeric enzymes with subunit sizes ranging within 17–28 kDa. GSTs have been purified from more than 24 individual insect species and, as with plants and mammals, the enzymes from insects are expressed at high levels, in multiple isoforms and in a different pattern depending on the developmental stage [4]. There are more than 30 GST genes in mosquitoes [4]. GSTs are also involved in many other intracellular processes, including protecting against oxidative stress, transporting intracellular compounds, catalyzing essential steps in biosynthetic pathways, and acting as signalling molecules [5].

GSTs have a higher affinity towards GSH because GSH is present at high intracellular concentrations. This means that the GSH binding site of GST may always be occupied [6] by this thiol. The active site residue in the N-terminal domain interacts with and activates the sulfhydryl group of GSH. In the delta and epsilon insect GST classes, this role is performed by a serine residue [7] and in most mammalian GSTs the active site residue is tyrosine [8]. Other reactions catalyzed by GSTs are reduction, addition, denitrosation, and thiolysis [9]. In insects, loci gene duplications, particularly within the insect specific delta and epsilon, have resulted in expansion of the GST family [7]. Two GST genes are alternatively spliced in* Anopheles gambiae* and four distinct peptides with differing catalytic properties are generated from the delta class causing much more diversity in* Anopheles* mosquito GSTs [10].

Insects GSTs are classified into six classes, sigma, epsilon, delta, theta, zeta, and kappa, by comparative analysis of* Drosophila melanogaster* and* Anopheles gambiae* genomes [13]. Delta and epsilon GST classes have expanded independently in* D. melanogaster* and* A. gambiae* suggesting that these enzymes play important roles in the adaptation of these species to their specific environment [5]. Insect GSTs are of great interest because of their potential involvement in insecticide resistance. For example, in the diamond back moth,* Plutella xylostella*, rising levels of an epsilon class GST confer resistance to organophosphate insecticide [14–16]. In the brown planthopper,* Nilaparvata lugens*, permethrin resistance is associated with the overexpression of nlGST1-1 [17].

**Figure 1:**
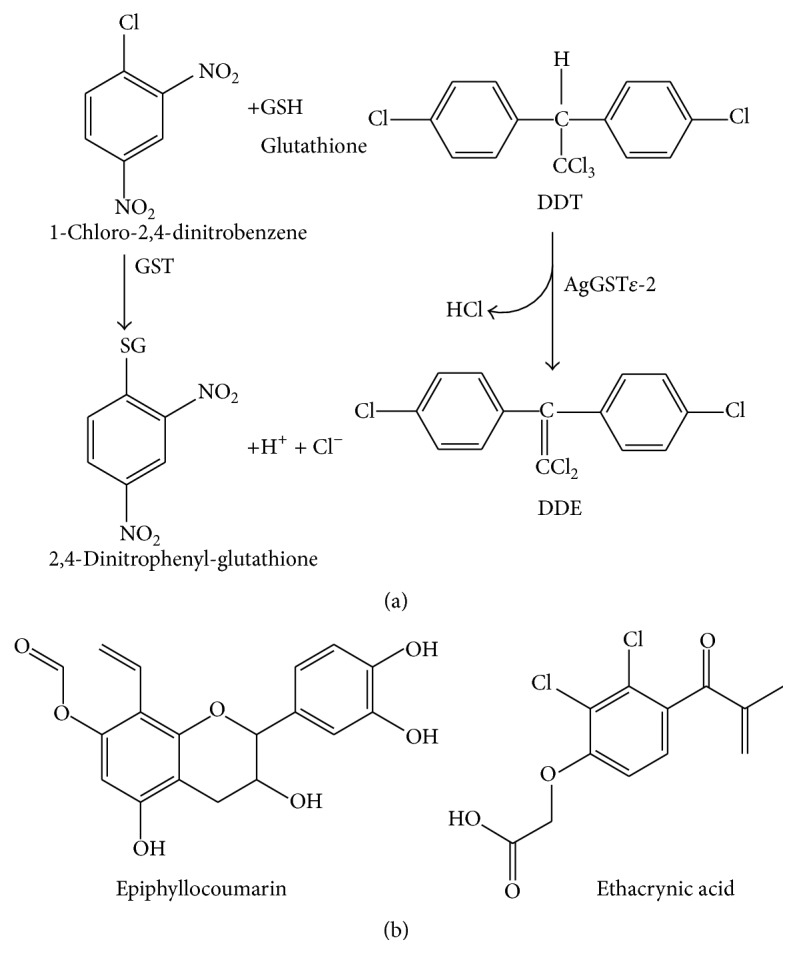
The conjugation reaction of 1-chloro-2,4-dinitrobenzene and DDT (a) as catalyzed by GSTs and (b) the compounds used in this study for the determination of inactivation properties of AgGST*ε*-2.

The epsilon class and the delta class have been implicated in detoxification, particularly in conferring resistance towards various insecticides [18]. In the mosquito,* A. gambiae*, increased expression of an epsilon class GST (GST*ε*) confers resistance to dichlorodiphenyltrichloroethane (DDT) [19]. The gene encoding this enzyme is one of the clusters of eight epsilon GST genes, arranged sequentially within 10.5 kb of DNA on division 33B of chromosome 3R [13]. These genes are arranged in close association with the DDT resistance locus rtdl [20]. Insecticide resistance mechanisms have a biochemical basis. The two major forms of biochemical resistance are target-site resistance and detoxification enzyme-based resistance [21]. DDT resistance in the mosquito is conferred by GSTs, particularly AgGST*ε*2 which is capable of metabolizing DDT to nontoxic metabolites 1,1-dichloro-2-(2-chlorophenyl)-2-(4-chlorophenyl)ethane (DDD) and 1,1-dichloro-2,2-bis-(p-chlorophenyl)ethane (DDE) which are easily excreted (Figure 1).

*Anopheles gambiae* refers to a complex of morphologically indistinguishable mosquitoes in the* Anopheles* genus, which contain the most important vectors of malaria in Sub-Saharan Africa and the most efficient malaria vectors in the world [22]. Species include* Anopheles arabiensis*,* Anopheles bwambae*,* Anopheles merus*,* Anopheles melas*,* Anopheles quadriannulatus*, and* Anopheles gambiae sensu stricto* [23]. The mosquito* Anopheles gambiae* is the principal vector of malaria in Africa. According to the latest WHO statistics, this parasitic disease infects from 300 to 500 million persons per year in the world and kills more than a million and a half each year, mainly African children [24]. DDT has potent insecticidal properties; it kills by opening sodium ion channels in insect neurons, causing the neuron to fire spontaneously [25]. This leads to spasms and eventual death. Previously, reports suggested that Tral 1 reversibly inhibited GST*ε*2* in vitro* [26] and so our aim was to determine if Tral 1 can also irreversibly inhibit GST*ε*2, resulting in time-dependent effects. These effects would be useful in controlling insecticide resistance in malarial mosquitoes that overexpress this enzyme.

## 2. Materials and Methods

### 2.1. Materials

All of the chemicals used, unless otherwise stated, were sourced from the Sigma-Aldrich Chemical Company (St. Louis, MO, USA). The natural plant product epiphyllocoumarin (Tral 1) was a gift from Professor Berhanu Abegaz from the Department of Chemistry, University of Botswana. Tral 1 was extracted from* Garcinia* species. These compounds were determined to be greater than 99% pure by liquid chromatography-mass spectroscopy and high performance liquid chromatography techniques. The* Escherichia coli* strain, BL21 (DE3) pLysS, was a gift from Dr. Hillary Ranson from the Liverpool School of Tropical Medicine (Pembroke Place Liverpool, UK). The cells were already transfected with a pET3a plasmid vector that contained the* AgGSTε2* gene.

### 2.2. Methods

#### 2.2.1. Expression and Purification of Recombinant AgGST*ε*2

A 100 mL starter culture of 2TYA medium, which is composed of 2 g tryptone, 1.5 g yeast extract, 0.5 g sodium chloride, and 1 g glycerol in 100 mL of distilled water, was prepared. The medium was autoclaved and cooled and 13.5 *μ*L of ampicillin (1 M stock) was added. The inoculating loop was sterilized by flaming before using it to enter culture material. An aliquot of the* E. coli* cells stab culture was taken using the loop and inoculated into the autoclaved media. The culture was incubated in a shaking incubator (Labcon, Labotec, South Africa) operating at 170 rpm and 37°C overnight for 20 hours. A 50% (v/v) glycerol solution was prepared in which 0.7 mL of the microbial broth and 0.3 mL of glycerol solution were placed in a cryopreservation microtube. The mixture was mixed and stored at −80°C. Three 2000 mL conical flasks containing 500 mL of 2TYA medium and 67.5 *μ*L of ampicillin (1 M stock) were inoculated with 5 *µ*L of the culture and incubated in a shaking incubator at the same settings. Another 500 mL volume of 2TYA media was used as the control. The absorbance of the inoculated media was measured at 600 nm using the UV-Visible 1601 spectrophotometer (Shimadzu UV-1601, Japan) against the control medium, which was used as a blank. The cells contained a gene for ampicillin resistance so ampicillin was added as a selectable marker. The absorbance was measured at 600 nm every 30 minutes until absorbance of 0.2–0.3 was obtained. Isopropylthiogalactoside (IPTG) was added to a final concentration of 0.2 mM in each flask to induce the expression of the* AgGSTε2* gene and the cells were grown overnight.

The bacteria were collected by centrifugation at 3500 rpm for 10 minutes at 15°C (3 K 15 Sigma Nr 19776, Laborzentrifugen, Germany). The volume of the cells was measured followed by the addition of an equal volume of lysis buffer (10 mM Tris-HCl pH7.8, 1 mM EDTA, 15% (w/v) glucose, 0.2% sodium azide, and 0.2 mM DTT). Chicken egg-white lysozyme was added to a final concentration of 1 mg/mL. The mixture was kept on ice for 1 hour with the occasional swirl to equally distribute the buffer around the cells. The cells were disrupted by sonication for 3 times (Dawe Soniprobe, England) at a setting of 5 for 30 seconds on ice. Phenylmethylsulphonylfluoride (PMSF) was added to a final concentration of 170 *μ*M to inhibit proteases. Cellular debris was removed by centrifugation at 105 000 ×g for 1 hour using a 50 Ti rotor in an ultracentrifuge (LE-80K Centrifuge, Beckman, USA) to obtain the cell supernatant fraction.

Affinity chromatography was used to purify AgGST*ε*2 using a combination of two matrix bound ligands hexyl-glutathione/glutathione (hexyl-GSH/GSH). Epoxy activated Sepharose 6B (Pharmacia Biotech, Uppsala, Sweden) affinity gel was used for affinity chromatography purification of AgGST*ε*2 as described before [26]. In order to determine the tubes in which most of the enzyme was located, a 1-chloro-2,4-dinitrobenzene (CDNB) assay was carried out. The reaction mixture contained sodium phosphate buffer (0.1 M, 1 mM EDTA, pH 6.5), AgGST*ε*2, and GSH. The GSH was added to a final concentration of 1 mM and the reaction was initiated by addition of CDNB whose final concentration was 1 mM. The increase in absorbance was recorded at 340 nm over a 60-second period. The kinetics mode of the UV-Visible 1601 spectrophotometer (Shimadzu UV-1601, Japan) was used. The fractions that exhibited activity were pooled and concentrated using Vivapore 10/20 solvent absorption concentrator with a molecular weight cut-off of 7500 daltons (7.5 kDa). Contaminants with molecular weights less than 7500 daltons would be removed together with water resulting in simultaneous concentration and purification of the enzyme.

The concentrated protein was dialyzed using a membrane with a molecular weight cut-off of 12 000 daltons against 9 L of buffer A (10 mM Tris-HCl pH 7.8, 1 mM EDTA, 0.2% sodium azide, and 0.2 mM DTT) using 3 × 3 L buffer changes. The various fractions collected during purification, that is, the cell supernatant, nonbound, affinity pool, and dialysate fractions, were tested for GST activity [26]. The 1 mL reaction mixture consisted of 1 mM glutathione (GSH), 1 mM CDNB, 1 mM EDTA, and 0.1 M sodium phosphate buffer at pH 6.5. The rate of conjugation of CDNB with GSH was determined kinetically at 340 nm, because the glutathione conjugate formed, 2,4-dinitrophenylglutathione (DNP-SG), absorbs maximally at 340 nm [27].

#### 2.2.2. Characterisation of AgGST*ε*2

The protein contents of the supernatant, nonbound, affinity pool, concentrate, and dialysate fractions, were measured by the Lowry method [12] using bovine serum albumin (BSA) as a standard. SDS PAGE was done in order to check for purity and to determine the molecular weight of the AgGST*ε*2. The purified protein, AgGST*ε*2, was subjected to polyacrylamide gel electrophoresis in the presence of 0.1% sodium dodecyl sulphate (SDS) according to the method of Laemmli [28]. SDS PAGE was done using 15% vertical slab gels and corun with a standard GST and Sigma low-range molecular weight markers, apoprotinin, *α*-lactalbumin, trypsin inhibitor, trypsinogen, bovine albumin, carbonic anhydrase, glyceraldehydes-3-phosphate dehydrogenase, ovalbumin, and albumin ranging from 6500 to 66000 Da (Sigma, St. Louis, MO, USA) using a BioRad Protean system (Biorad Laboratories, California, USA). The protein bands were stained using Coomassie G Stain (0.025% Coomassie G250, 40% methanol, and 7% acetic acid) overnight, destained for 1 hr in 50% methanol and 10% acetic acid, and then further destained in 5% methanol and 7% acetic acid solution. The gel picture was taken using a gel documentation station Minibis Bioimaging System (Minibis Bioimaging Systems, DNR Bioimaging System, Israel).

#### 2.2.3. Determination of Enzyme Inactivation by Tral 1

Incubation mixtures contained AgGST*ε*2 (final concentration 0.0625 *μ*M) and 0.2 M potassium phosphate buffer pH 7.4 with 0.2 mM EDTA together with the 25 *µ*M of the test compound Tral 1. The incubation temperature was 30°C. At fixed time intervals, 67 *µ*L of the incubation mixture was withdrawn and assayed for GST activity. These incubations were run in parallel with positive and negative controls. The negative control contained AgGST*ε*2 and buffer. The positive control contained AgGST*ε*2, buffer, and 25 *µ*M ethacrynic acid. The inactivation parameter *t*_1/2_ (the half-life) was obtained by plotting graphs of the percentage remaining enzyme activity with time. To investigate concentration-dependent inactivation, incubations were set up as for time-dependent inactivation but with varying concentrations of inhibitor. After a fixed period of preincubation, an aliquot of the incubation mixture was withdrawn and assayed for GST activity. The inactivation parameters *k*_inact_ and *K*_I_ of the compound with AgGST*ε*2 were obtained by analysing the data using the equation (see [11])

(1) $$\begin{array}{c} \frac{\left[\ln\left(E/E_{o}\right)\right]}{t}=-\left(\;\frac{k_{\text{inact}}\cdot \left[\text{I}\right]}{\left[\text{I}\right]+\left[K_{\text{I}}\right]}\;\right), \end{array}$$

where *E*_*o*_ is enzyme activity of the control (no inhibition) and *E* is enzyme activity following incubation for a certain time. This equation is analogous to the Michaelis-Menten equation. Therefore, if nonlinear regression is performed, the maximum *Y* (*Y*_max⁡_) value will be equal to *k*_inact_, and the concentration of inhibitor giving half *Y*_max⁡_ will be equal to *K*_I_. The raw data were transformed and transform values were fit to the Michaelis-Menten curve using GraphPad*™* version 4.00 for windows (GraphPad Software Inc.).

#### 2.2.4. Statistical Analyses

The values obtained were expressed as mean ± standard deviation and the results were compared against control by using one-way ANOVA. Dunnett's Multiple Comparison Test was used as the posttest using GraphPad InStat software. Differences between the means with *P* values of 0.05 or less were considered significant.

## 3. Results

### 3.1. Purification of GST*ε*2

**Figure 2:**
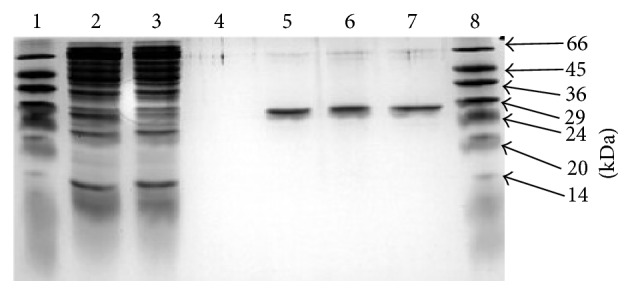
Electrophoretic analysis of the different fractions of AgGST*ε*2. Lane 1 contained molecular weight markers. Lane 2 contained the supernatant fraction obtained after lysis, sonication, and centrifugation of* E. coli* cells. Lane 3 contained the nonbound fraction containing protein that did not bind the affinity chromatographic ligands hexyl GSH/GSH. Lane 4 contained the wash fraction. Lane 5 contains the affinity pool fraction. The concentrate fraction is contained in lane 6 whilst lane 7 contained the dialysate fraction. Lanes 2 and 3 contained numerous bands indicating the presence of unwanted proteins. The single bands shown in lanes 5, 6, and 7 indicate that GST*ε*2 was purified to homogeneity.

Table 1 shows the results obtained for purification of AgGST*ε*2 from the crude cell extracts of cell lysate to the concentrated protein. As the protein was being purified, the specific activity which is a measure of the purity of protein increased from 0.52 *μ*mol min^−1^ mg^−1^ for the crude protein to 46 *µ*mol min^−1^ mg^−1^ for the dialysate fraction with an overall yield of 40% and a fold purity of 86.2. The protein content and percentage yield continued to decrease with each successive purification step indicating a progressive loss of protein at each purification step while the purification fold increases. The nonbound fraction had the highest total protein compared to other purification stages besides the supernatant. The wash fraction had the lowest specific activity of 0.012 *μ*mol min^−1^ mg^−1^ because all of the protein had now been bound to the matrix and the nonbound fraction eluted. The specific activity of AgGST*ε*2 is also comparable with the activities described for other recombinant AgGST*ε*2 of the same class, for example, the specific activity of AgGST*ε*1 with CDNB as a substrate was reported to be 56.4 *μ*mol min^−1^ mg^−1^ [19]. The molecular weight of AgGST*ε*2 obtained was 25 kDa (Figure 2), a value which is the same as the literature value [26].

### 3.2. Determination of Inhibition of AGST*ε*2 by Tral 1

**Figure 3:**
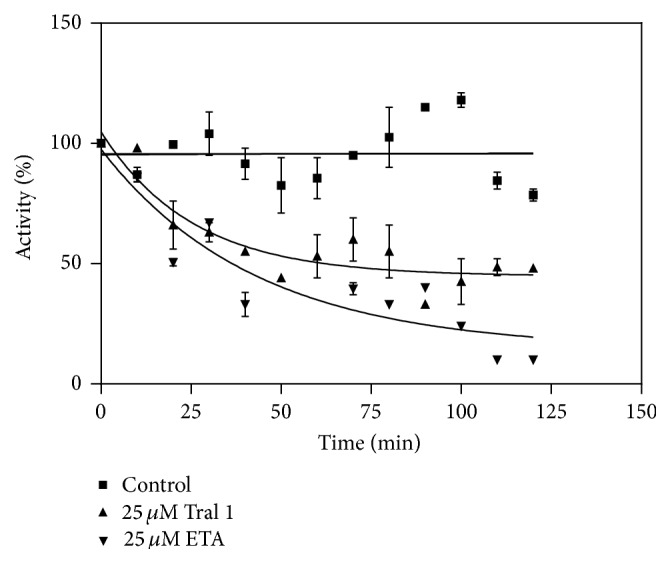
Percentage activity against time for the time-dependent inactivation of AgGST*ε*2 by Tral 1 and ETA. The activity was determined every 15 min over a 2 h period. The *t*_1/2_ was determined by nonlinear regression.

**Figure 4:**
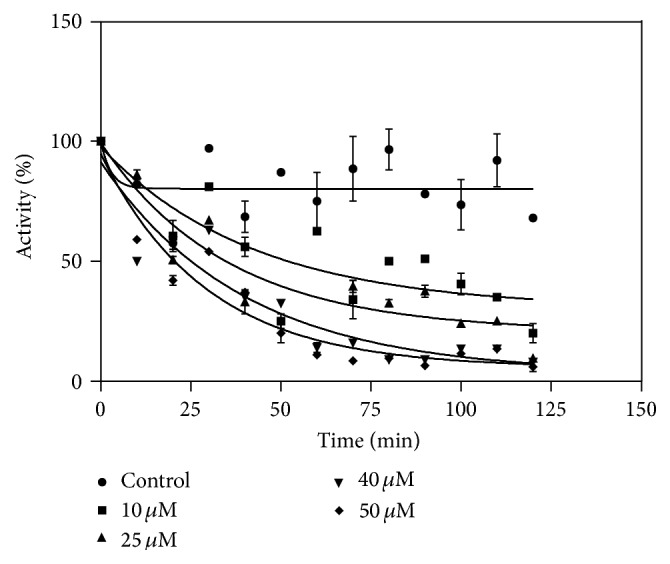
Percentage activity against time for the concentration-dependent inactivation of GST*ε*2 by Tral 1. The activity was determined every 15 min over a 2 h period. The *t*_1/2_ was determined by nonlinear regression.

**Figure 5:**
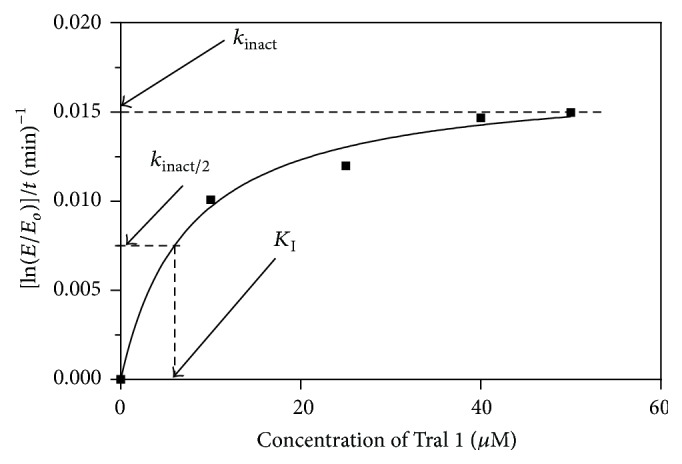
The rate of loss of GST*ε*2 activity with increase in Tral 1 concentration after 30 min incubation. This graph was obtained by analysing data according to the method of Maurer and Fung [11] so as to determine the inactivation parameters. *K*_I_ is the concentration of inhibitor which gives half the maximal rate of inactivation.

The purified AgGST*ε*2 was exposed to 25 *μ*M of Tral 1 and it was found that at this concentration Tral 1 inactivated AgGST*ε*2 in a time-dependent manner. The positive control ETA also inactivated AgGST*ε*2 in a time-dependent manner. The inactivation rates were expressed as half-life (*t*_1/2_). The half-life for Tral 1 and ETA was obtained from graphs such as that shown in Figure 3. The half-life of Tral 1 was 30 minutes and that of ETA was 22 minutes as shown in Figure 3, indicating that Tral 1 is a potent inactivator of AgGST*ε*2 but not as potent as ETA. These half-lives were obtained after exposing the enzyme to 25 *μ*M of Tral 1 and ETA. The purified AgGST*ε*2 was exposed to various concentrations of the inhibitor (10 *μ*M, 25 *μ*M, 40 *μ*M, and 50 *μ*M). The results showed that Tral 1 also inactivated AgGST*ε*2 in a concentration-dependent manner (Figure 4). The inactivation parameters *k*_inact_ and *K*_I_ of Tral 1 with GST*ε*2 were determined from plots such as Figure 5, *k*_inact_ being the maximum *Y* value and *K*_I_ being the concentration of the natural product at half the maximum *Y* value. The half-lives of Tral 1 at these various concentrations were also obtained and are shown in Table 2 showing us the effect of increasing concentration. As the concentration of the inhibitor Tral 1 increases the half-life decreases meaning the half-life is inversely dependent on the concentration of the inhibitor. The* in vitro* characterisation of a mechanism-based enzyme inactivator includes the determination of the maximum inactivation rate constant (*k*_inact_) and the inactivator concentration that produces half-maximal rate of inactivation (*K*_I_). The inactivation parameters* in vitro k*_inact_ and *K*_I_ were found to be 0.02 ± 0.0011 min^−1^ and 7.5 ± 2.1 *µ*M, respectively, after 90 minutes of incubation.

## 4. Discussion

Compounds that bind irreversibly to a protein usually form covalent bonds that can modify the active site of an enzyme. This type of inhibition is called irreversible inhibition, and usually the inhibitor contains highly reactive functional groups such as epoxides, aldehydes, halogenoalkanes, or alkenes [29]. Coumarins are known to irreversibly inhibit GSTs by forming a reactive epoxide [2]. Other coumarins have been found to inactivate GSTs by covalent modification of an essential amino acid. In the delta and epsilon insect GSTs, the active site residue is serine [30]. Thus, it could also be postulated that Tral 1 inactivates AgGST*ε*2 by covalent binding to the highly reactive serine residue. The binding and inactivation steps were investigated by incubating AgGST*ε*2 and Tral 1 and then assaying the amount of activity remaining over time. The activity of AgGST*ε*2 decreased in a time-dependent manner and concentration-dependent manner. Thus, AgGST*ε*2 could be sensitized to the effects of DDT by cospraying the insecticide with Tral 1. Other studies have shown that inhibition of human GSTs by ethacrynic acid can sensitize human cancer cells to the effects of anticancer agents such as melphalan [31].

The data for the enzyme alone indicated that the enzyme was stable and any change noted from the one with Tral 1 is not due to enzyme degradation.

**Figure 6:**
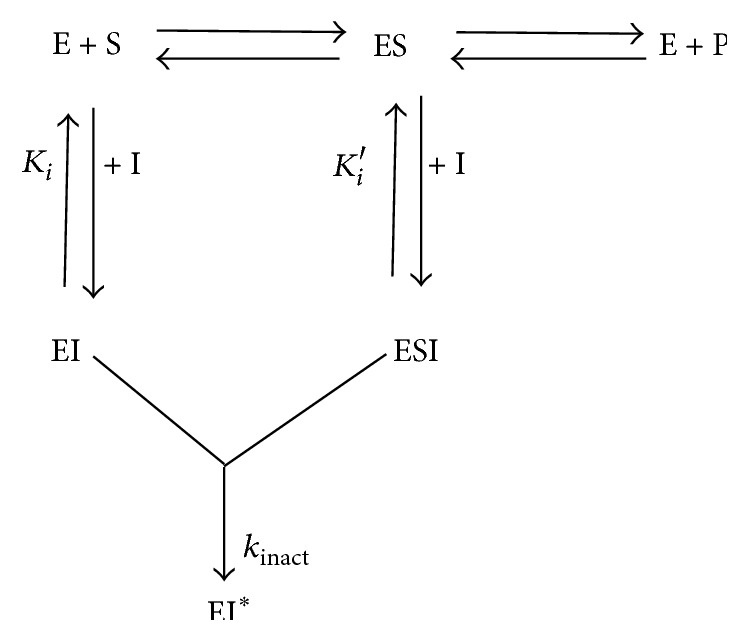
Pathways for irreversible inhibition [11]. *K*_i_ is a measure of the binding affinity for the inactivator for the free enzyme. *K*_i_′ is a measure of the binding affinity for the inactivator for the enzyme-substrate complex and *k*_inact_ describes the rate at which inhibitor-enzyme complex is irreversibly transformed into EI^*∗*^.

When a compound binds irreversibly to an enzyme, there is formation of an enzyme-inhibitor complex (EI) or an enzyme-substrate inhibitor complex (ESI). The covalently modified enzyme cannot produce a product (Figure 6); rather it produces a “dead-end complex” EI^*∗*^ [11]. The time required to form the EI^*∗*^ is called the inactivation rate or *k*_inact_. In order to get this value, the enzyme is incubated with a compound so that it binds and inactivates it. The amount of activity remaining over time is determined. If the compound is an inactivator, there will be a time-dependent decrease in activity and rate of inactivation would follow an exponential decay. If the formation of EI^*∗*^ is reversible, the inactivation rate will be saturable enabling the calculation of *k*_inact_ and *K*_I_ from it [11]. The inactivation parameters *k*_inact_ and *K*_I_ were found to be 0.02 ± 0.0011 min^−1^ and 7.5 ± 2.1 *µ*M, respectively, after 90 minutes of incubation as shown in Table 2. Diospyrin, a natural plant product, was shown to irreversibly inhibit GSTP1-1 giving a *K*_I_ value of 0.7 ± 0.2 *μ*M [32]. Comparing this value with the one obtained for AgGST*ε*2 suggests that diospyrin requires a lesser concentration to give the half-maximal rate of inactivation as compared to Tral 1. We have also previously shown that Tral-1 inhibited AgGST*ε*2 reversibly showing noncompetitive and competitive inhibition with respect to GSH and CDNB [26]. The new results suggest that Tral-1 can reversibly and irreversibly inhibit AgGST*ε*2.

Ethacrynic acid (ETA), a diuretic hormone, has been studied by several groups for its potential for reversible inhibition of GSTs. ETA was able to inhibit the GST pi class of rat, mouse, or man by reaction with cysteine 47 [2]. ETA is an unsaturated ketone derivative of an aryloxyacetic acid. *α*,*β*-Unsaturated aldehydes and ketones are one of the classes most intensively studied for their potency to irreversibly inhibit GSTs. For example, tetrachloro-1,4-benzoquinone and its particular GSH conjugate have been shown to inactivate rat GST isoforms [29]. The GSH conjugate increased the rate of inactivation as compared to the parent quinone. For ETA, it can also be postulated that the formation of GSH conjugate increased the rate of inactivation. ETA-GSH conjugate is formed enzymatically and nonenzymatically and itself is an inhibitor of GST [33]. Loss of activity is also due to the action of ETA.

ETA has been shown to inhibit GSTP1-1 in a time-dependent manner at concentrations as low as 25 *μ*M [34]. Inhibition of GST P1-1 has been postulated to be due to the presence of *α*-*β*-unsaturated carbonyl derivatives that bind covalently to the cysteine 47 in the active site of the enzyme. In other studies of ETA with GST P1-1, the half-life obtained was 20 minutes [35]. In this study, the half-life obtained was comparable at 22 minutes. The results show that ETA is a potent inhibitor of GSTs. Other plant natural compounds have also been shown to be effective irreversible inhibitors of GSTs. GSTs M1-1, M2-2, and P1-1 were inactivated by the two polyphenolic compounds ellagic acid and curcumin [34].

Glutathione transferases (GSTs) are important detoxifying enzymes. In the malaria vector* Anopheles gambiae*, elevated GSTs are associated with resistance to the organochlorine insecticide DDT. AgGST*ε*2 has been implicated in conferring this resistance [36]. The results obtained in this study indicate that Tral 1 is a potent inactivator of GST*ε*2. Tral 1 was found to be an irreversible inactivator of GST*ε*2 both in a time-dependent manner and in a concentration-dependent manner. Given these inhibitory properties, Tral 1 may be a potent GST chemomodulator and could be used to reverse DDT resistance. ETA was found to be a more effective irreversible inhibitor of the enzyme as compared to Tral 1. However, combined with our previous finding [26], Tral 1 acts in a similar manner to ethacrynic acid on GSTs where it inhibits the enzyme directly and/or in a time-dependent manner.

## Figures and Tables

**Table 1:** Summary of the purification of recombinant *Anopheles gambiae *glutathione-S-transferase epsilon 2.

Sample	Total activity (units)	Total protein (mg)	Specific activity (units mg^−1^)	Percentage yield (%)	Fold purity
Supernatant	225	432	0.52	100	1
Nonbound	135	320	0.42	60	0.8
Wash	2.2	104	0.012	1	0.02
Affinity pool	67	11	6.08	26	11.7
Concentrate	76	3	27	36	51.1
Dialysate	81	1.3	46	36	86.2

The activity of the enzyme is expressed per milligram of total protein (expressed in *μ*mol min^−1^ mg^−1^). Protein was determined by the method of Lowry et al. [12].

**Table 2:** Inactivation rates expressed as half-life for the concentration-dependent inactivation.

Tral 1 (*μ*M)	Half-life (minutes)	Kinetic parameter	
10	42	*k*_inact_	0.02 ± 0.0011 min^−1^
25	33	*K*_I_	7.5 ± 2.1 *μ*M
40	24		
50	13		

## Abbreviations

DDT: Dichlorodiphenyltrichloroethane
GSTs: Glutathione transferases
GSH: Reduced glutathione
Tral 1: Epiphyllocoumarin
GST*ε*2: Glutathione transferase epsilon 2
CDNB: 1-Chloro-2,4-dinitrobenzene
*K*_I_: The concentration of inhibitor which gives half the maximal rate of inactivation
*k*_inact_: The maximal rate of enzyme inactivation at a saturating concentration of inhibitor
*K*_i_: A measure of the binding affinity for the inactivator for the free enzyme
*K*_i_′: A measure of the binding affinity for the inactivator for the enzyme-substrate complex.
